# The potential probiotic *Lactobacillus rhamnosus* CNCM I-3690 strain protects the intestinal barrier by stimulating both mucus production and cytoprotective response

**DOI:** 10.1038/s41598-019-41738-5

**Published:** 2019-04-01

**Authors:** Rebeca Martín, Celia Chamignon, Nadia Mhedbi-Hajri, Florian Chain, Muriel Derrien, Unai Escribano-Vázquez, Peggy Garault, Aurélie Cotillard, Hang Phuong Pham, Christian Chervaux, Luis G. Bermúdez-Humarán, Tamara Smokvina, Philippe Langella

**Affiliations:** 1 INRA, Commensal and Probiotics-Host Interactions Laboratory, Micalis Institute, INRA, AgroParisTech, Université Paris-Saclay, 78350 Jouy-en-Josas, France; 2 Danone Nutricia Research, Av de la Vauve, 91767 Palaiseau, France; 3 ILTOO Pharma, 14 Rue des Reculettes, 75013 Paris, France

## Abstract

The gut barrier plays an important role in human health. When barrier function is impaired, altered permeability and barrier dysfunction can occur, leading to inflammatory bowel diseases, irritable bowel syndrome or obesity. Several bacteria, including pathogens and commensals, have been found to directly or indirectly modulate intestinal barrier function. The use of probiotic strains could be an important landmark in the management of gut dysfunction with a clear impact on the general population. Previously, we found that *Lactobacillus rhamnosus* CNCM I-3690 can protect intestinal barrier functions in mice inflammation model. Here, we investigated its mechanism of action. Our results show that CNCM I-3690 can (i) physically maintain modulated goblet cells and the mucus layer and (ii) counteract changes in local and systemic lymphocytes. Furthermore, mice colonic transcriptome analysis revealed that CNCM I-3690 enhances the expression of genes related to healthy gut permeability: motility and absorption, cell proliferation; and protective functions by inhibiting endogenous proteases. Finally, SpaFED pili are clearly important effectors since an *L. rhamnosus ΔspaF* mutant failed to provide the same benefits as the wild type strain. Taken together, our data suggest that CNCM I-3690 restores impaired intestinal barrier functions via anti-inflammatory and cytoprotective responses.

## Introduction

The genus *Lactobacillus* is a phylogenetically diverse group of Gram-positive bacteria. It includes more than 200 species found in diverse ecosystems, including the human body and fermented dairy products^1^. *Lactobacillus rhamnosus* is an anaerobic facultative heterofermentative rod-shaped bacterium that can live in different parts of the human body, including the gastrointestinal tract (GIT)^2^. Some lactobacilli strains, including several *L. rhamnosus*, are potential probiotics as they can maintain gut homeostasis^3^ and relieve dysbiosis-related diseases^4^. At present, *L. rhamnosus* GG (LGG) is one of the most studied and characterized probiotic strains^5^. Indeed, it can provide numerous beneficial effects, as seen in *in vitro* and *in vivo* models and in humans^5,6^.

Some lactobacilli can adhere to mammalian tissues, a key feature that allows adaptation to the GIT, crosstalk with the host and competitive exclusion of pathogens^7–9^. Thanks to the close relationships established with their hosts, some probiotic strains can provide additional benefits: for example, they can mediate either immune responses or barrier functions^10^. Therefore, among probiotics, GIT adhesion is often a crucial feature. Recently, several studies have sought to identify adhesion proteins as well as the mechanisms underlying adhesion^11,12^. For instance, a functional analysis of LGG revealed that SpaCBA pili, encoded by the *spaCBA* operon, play a key role in adhesion and immunomodulation^13–15^.

The intestinal barrier separates the self from the non-self and serves as the first line of defence against external threats such as toxins and pathogens. It presents a functional unit of a physical barrier consisting of a mucus layer and a monolayer of epithelial cells and of a mucosal lymphoid system that together efficiently discriminate between pathogenic and commensal microorganisms^16^. When the intestinal barrier is healthy, it allows selective paracellular transport of nutrients, regulating solute and water fluxes while preventing the entry of bacteria and toxins. When barrier function is impaired, altered permeability and dysfunction can result, ultimately leading to problems such as irritable bowel syndrome (IBS), food allergies and obesity^17,18^. Different bacteria, including pathogens and commensals, can directly or indirectly modulate intestinal barrier function. For instance, LGG, *Escherichia coli* Nissle 1917, and a commercial mixture of lactobacilli and bifidobacteria (VSL#3) have been shown to prevent “leaky gut” by enhancing mucosal integrity and decreasing barrier permeability^19–21^.

Previously, we reported that *L. rhamnosus* CNCM I-3690 counteracts the increased intestinal permeability induced by mild inflammation as efficiently as the commensal *Faecalibacterium prausnitzii* A2-165^22^. Furthermore, this strain protects against oxidative stress in *Caenorhabditis elegans*^23^. Here, we aimed to decipher the mechanisms underlying *L. rhamnosus* CNCM I-3690’s effects on gut barrier and identify the bacterial effectors involved.

## Results

### Adhesins present in *L. rhamnosus* CNCM I-3690

**Figure 1:**
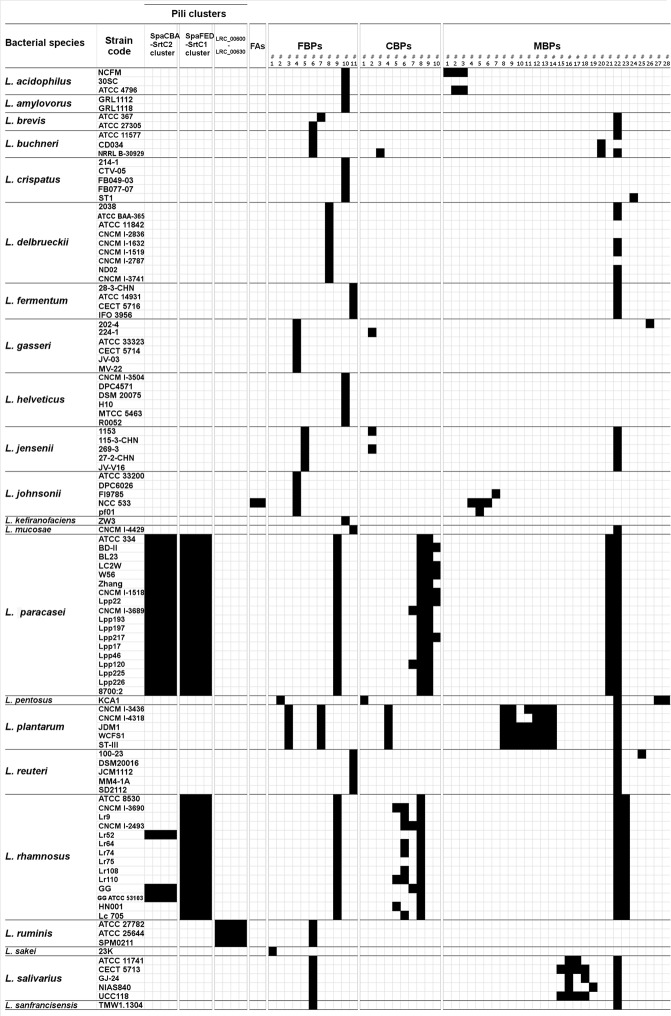
Distribution of 63 adhesin proteins among 105 strains of 23 *Lactobacillus* species. The presence (black squares) or absence (white squares) of an adhesin’s ortholog was determined using BLASTP. The list is not exhaustive and comprises fimbrial adhesins (FAs), fibronectin-binding proteins (FBPs), collagen-binding proteins (CBPs), and mucus-binding proteins (MBPs).

We studied adhesins which are believed to play a crucial role in the persistence of lactobacilli strains in the digestive tract. In particular, we determined which of 63 adhesin proteins found in lactobacilli were present in *L. rhamnosus* CNCM I-3690 (Fig. 1).

Two pilus-related operons (*spaCBA* and *spaFED*) containing specific sortase-encoding genes (*srtC2* and *srtC1*, respectively) were found in *L. rhamnosus* (Fig. 1). The *spaFED* operon has been observed in all *L. rhamnosus* strains analyzed, including CNCM I-3690. In contrast, the *spaCBA* operon was found only in *L. rhamnosus* GG and two other closely related strains (Lr52 and ATCC53103). *L. rhamnosus spaCBA* operon shows high similarity to that found in all *L. paracasei* strains tested. Other pilus-related cluster (LRC_00600-LRC_00630), identified in *L. ruminis* ATCC 27782^24^, or two fibrial adhesins (FAs), of *L. johnsonii* NCC 533^25^ were all absent from CNCM I-3690 (Fig. 1). However, we observed a Fibronectin-Binding Proteins (FBPs) common in both *L. paracasei* and *L. rhamnosus* (Fig. 1). In addition, three out of four Choline-Binding Proteins (CBPs) found in *L. rhamnosus* were present in CNCM I-3690; (Fig. 1). Of the 28 Mucus-Binding Proteins (MBPs) examined, MBP#23 (containing four mucus-binding domains [Pfam-MucBP]) was present in all *L. rhamnosus* strains including CNCM I-3690. Similarly, MapA#22, widely distributed among lactobacilli, was detected in CNCM I-3690 (Fig. 1).

### The *ΔspaF* mutant lacks the anti-inflammatory, protective, and adhesive properties of the CNCM I-3690 wild type *in vitro*

**Figure 2:**
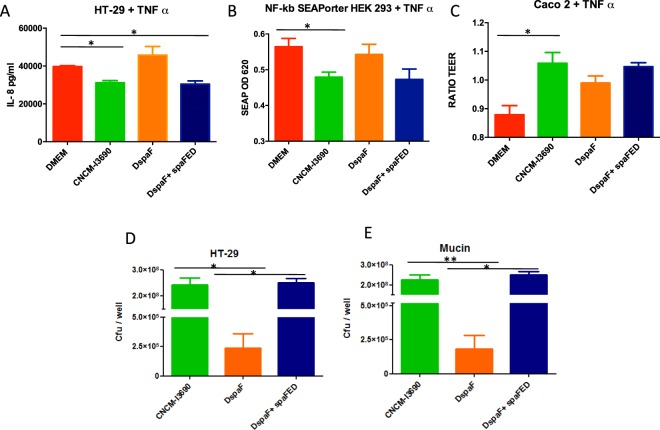
Immunomodulatory, protective, and adhesive properties of *L. rhamnosus* CNCM I-3690 in *in vitro* tests. Imunomodulatory tests with HT-29 and HEK/SeaPorter cells (**A**,**B**), trans epithelial resistance provided to Caco-2 cells (**C**), and adhesion to HT-29 cells and mucin (**D**,**E**). Significance: *p < 0.05 and **p < 0.01 (*n* = 3 × 3).

The anti-inflammatory properties of the CNCM I-3690 strain were confirmed *in vitro* using challenged HT-29 cells and or NF-κβ/SEAPorter HEK 293 cells (Fig. 2A,B). When HT-29 cells challenged with TNF-α were co-incubated with the CNCM I-3690 strain, there was a statistically significant decrease in IL-8 production. In similarly challenged NF-κβ/SEAPorter HEK 293 cells, NFκβ activation decreased following co-incubation with the CNCM I-3690. In both models, co-incubation with the *ΔspaF* mutant had no effect (Fig. 2A,B). To further determine if the CNCM I-3690 protects the barrier, we measured the trans-epithelial electrical resistance (TEER) of Caco-2 cells challenged with TNF-α (Fig. 2C). The protective effect was only significant for the wild type (WT) strain (Fig. 2C). When adhesion to HT-29 cells or mucin (Fig. 2D,E) was examined, the CNCM I-3690 was highly adhesive while the *ΔspaF* mutant was not (10^3^-fold less).

In all experiments, the complementation with *spaF* recovered the wild-type phenotype (Fig. 2).

### *L. rhamnosus* CNCM I-3690 can alleviate low-grade inflammation *in vivo*

**Figure 3:**
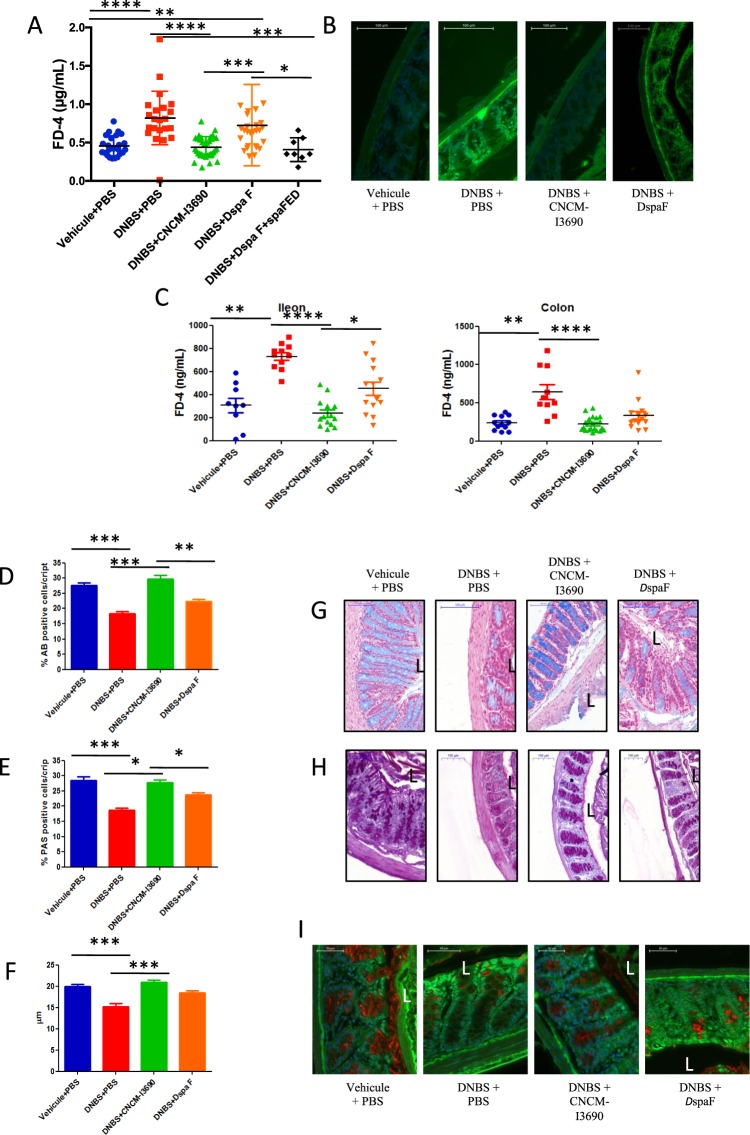
Effects of probiotic treatment on *in vivo* and *in vitro* gut permeability, goblet cell abundance, and mucus layer morphology. *In vivo* gut permeability in experimental mice (orally gavaged with FITC-dextran) (**A**,**B**). *In vitro* gut permeability for colon and ileum samples taken from experimental mice (*n* = 10 per group) (**C**). Representative photos and % of positive cells from AB-staining (**D**,**G**) and PAS (**E**,**H**) tests. Mucus layer thickness and representative photos of samples stained with MUC2 antibody (**F**,**I**) (*n* = 16). There were five groups of mice: control, untreated, treated with the *L. rhamnosus* CNCM I-3690 WT, and treated with *L. rhamnosus DspaF* mutant and treated with the complemented strain (*L. rhamnosus DspaF* + *spaFED*). Significance: *p < 0.05 and **p < 0.01.

We used a model of DNBS-induced chronic micro-inflammation. We confirmed the presence of low-grade inflammation and quantified it by measuring health and inflammatory parameters (Fig. S1B–F). There were slight differences among the experimental mice groups for all the metrics except weight loss (Fig. S1B). In particular, treatment with the CNCM I-3690 strain improved the colonic macroscopic scores (Fig. S1B), colonic cytokine levels (Fig. S1F), colon and ileum MPO activities (Fig. S1C,D) and *in vivo* permeability (Fig. 3A) while treatment with the *ΔspaF* mutant did not. Interestingly, treatment with WT, but not with *ΔspaF* mutant, significantly decreased the levels of IL-6, IFN-β, and IFN-γ (p < 0.05) and increased the level of IL-10 (Fig. S1F). Furthermore, while FD4 permeability was high in the untreated group, it did not do so in either the control group or the CNCM I-3690-treated group (microscopy results; Fig. 3B).

### *L. rhamnosus* CNCM I-3690 restores colon and ileum permeability

We quantified colon and ileal permeability *in vitro* using Ussing Chambers. The results showed similar patterns that in *in vivo* permeability tests (Fig. 3C). For both tissues, the untreated group had greater FD4 permeation, while FD4 permeation was similar for the control group and the CNCM I-3690-treated group (p < 0.05) (Fig. 3C). The permeability in both *in vitro* and *in vivo* remained increased in the *ΔspaF*-treated group (p < 0.05) (Fig. 3). However, this pattern was less dramatic in the colon than in ileum samples (Fig. 3C). Regarding the complemented strain, it recovered the WT phenotype *in vivo* (Fig. 3)

### *L. rhamnosus* CNCM I-3690 improves colonic barrier by increasing mucus production and restoring Goblet cells (GC) population

We analyzed the effect of CNCM I-3690 on the mucus layer and mucus producing cells. HES-stained cells showed no significant differences in general morphology among all the groups (data not shown). The numbers of GCs highlighted by Alcian blue (AB) staining (Fig. 3D,G) or the periodic acid-Schiff (PAS) method (Fig. 3E,H), which reveal the presence of acid or neutral mucopolysaccharides, respectively, were significantly lower in the untreated group than in the CNCM I-3690-treated group (p < 0.05). In the latter, numbers were similar to those for the control group. The *ΔspaF–treated* group showed limited staining, suggesting a loss of this ability (Fig. 3D,E).

Mucus layer thickness was measured via MUC2 immunohistochemistry (Fig. 3F,I). The untreated and the *ΔspaF–treated* groups had a thinner mucus layer than the control group or the CNCM I-3690-treated group (p < 0.05).

### *L. rhamnosus* CNCM I-3690 exhibits anti-inflammatory effects in the spleen and mesenteric lymph nodes (MLN)

**Figure 4:**
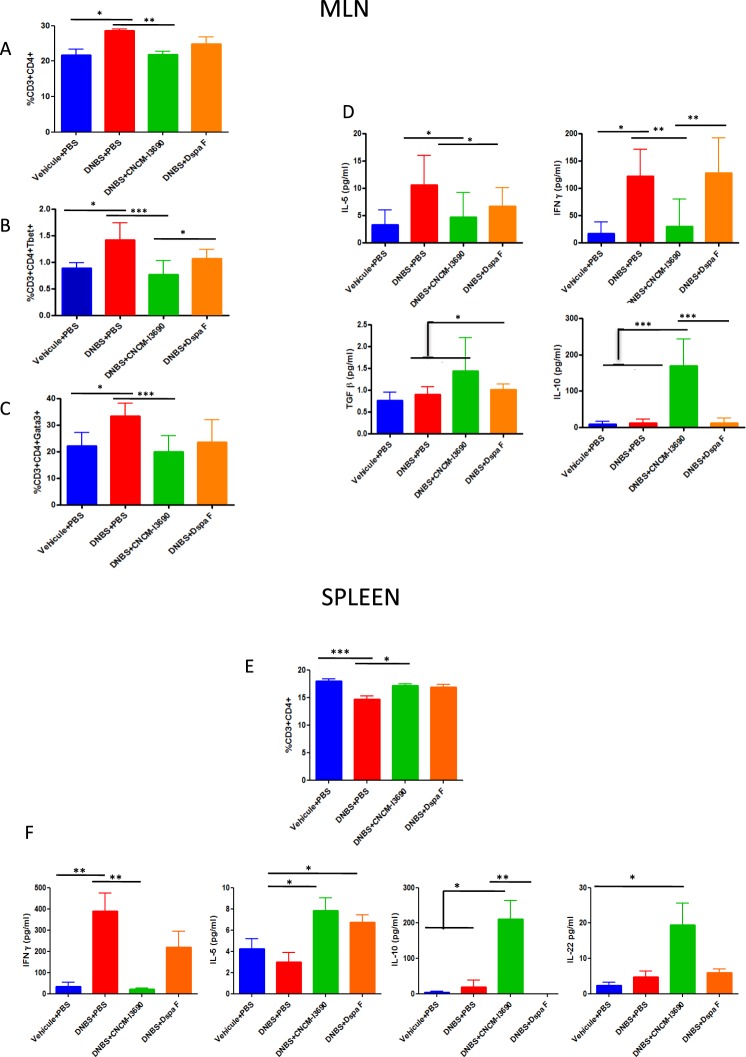
Immunological activity in MLN and spleen cells. MLN cells positive for CD3^+^, CD4^+^, T-bet, or GATA-3 as detected using flow cytometry (**A**–**C**), and cytokine production in MLN cells stimulated by CD3^+^/CD28^+^ (**D**). Spleen cells positive for CD3^+^/CD4^+^ as detected by flow cytometry (**E**), and cytokine production in spleen cells stimulated with CD3^+^/CD28^+^ (**E**). Mice groups and significance as in Fig. 3 (n = 8).

CD3^+^/CD4^+^ T cell percentages were higher in the MLNs (Fig. 4A) and lower in the spleen (Fig. 4E) for the untreated group than for the control group (p < 0.05). In CNCM I-3690-treated mice, cells percentages turn similar to control group (Fig. 4E). However, there were not such significant differences in *ΔspaF–treated* group (Fig. 4A,E). Regarding CD3^+^/CD8^+^ or CD3^+^/NK^+^ T cell percentages, no differences were found (data not shown). The T-bet and GATA-3 results show that, for the MLNs, Th1 and Th2 activity was higher in the untreated group and the *ΔspaF*-treated group than in the CNCM I-3690-treated group (p < 0.05) (Fig. 4B,C).

Isolated T cells from MLN and spleen cells were stimulated with CD28^+^/CD3^+^ (Fig. 4D,F). We measured cytokine levels in the culture supernatants (Fig. 4D,F). In the MLN cells from CNCM I-3096-treated mice, both Th1 and Th2 activity (using IFN-γ and IL-5 as proxies respectively) were relatively lower and T_reg_ levels (using IL-10 as a proxy) were relatively higher (Fig. 4D), revealing an anti-inflammatory response. In the spleen cells from CNCM I-3096-treated mice, Th1 activity was relatively lower but Th2 activity and T_reg_ levels were relatively higher. Thus, treatment with CNCM I-3690 could control the increase in IFNγ levels resulting from inflammation. In contrast, *ΔspaF–treated* group showed a similar patter to the non-treated group in both MLN and spleen samples.

### Transcriptomic analysis reveals that CNCM I-3690 up-regulates genes related to healthy gut permeability and protective functions

**Figure 5:**
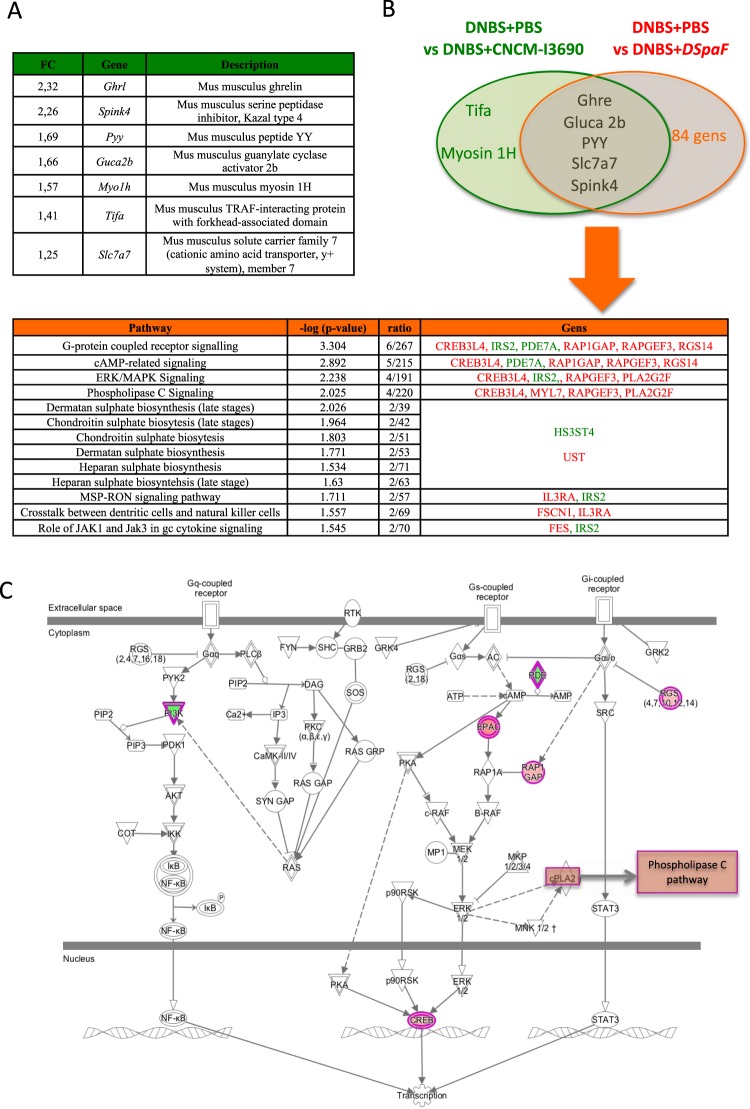
Colonic transcriptome analysis of mice treated with probiotics. Genes modulated by the CNCM I-3690 WT versus DNBS-PBS (**A**) Genes modulated by the CNCM I-3690 WT versus by the *DspaF* mutant and the major pathways modulated by *DspaF*mutant as identified via IPA (**B**). Representation of G-protein-coupled receptor signaling (including cAMP-related signaling), ERK/MAPK signaling, and phospholipase C signaling (identified and modified via IPA) (**C**). Genes upregulated and downregulated by the *DspaF*mutant are in red and green, respectively. Abbreviations: CREB3L4—cAMP responsive element-binding protein 3 like 4; IRS-2—insulin receptor substrate-2; PDE7A—phosphodiesterase 7A; RAP1GAP—RAP1 GTPase-activating protein; RAPGEF3—Rap guanine nucleotide exchange factor 3; RGS14—regulator of G-protein signaling 14; PLA2G2F—phospholipase A2 group IIF; MYL7—myosin light chain 7; HS3ST4—heparan sulfate glucosamine 3-O- sulfotransferase 4; UST—uronyl 2-sulfotransferase; IL3RA—interleukin-3 receptor; FSCN1—fascin actin-bundling protein 1; and FES—fes proto-oncogene.

The transcriptome analysis of colon samples at the endpoint from mice revealed that seven genes were differently expressed between the untreated and the CNCM I-3690-treated groups (Fig. 5A). Interestingly, the expression of 89 genes differed between the untreated and the *ΔspaF –treated* groups, including 5 genes that were also upregulated in the CNCM I-3690-treated group (Tables S1–S2 and Fig. 5B,C). The IPA of the specific signaling pathways modulated by the mutant reveals that *Δspa F* mutant was able to increase G-protein-coupled receptor signaling (especially cAMP-related signaling), as well as ERK/MAPK signaling and phospholipase C signaling (Fig. 5C). The *ΔspaF* mutant was also able to affect glycosaminoglycan (GAG) synthesis. RT-qPCR was carried out on a selection of seven genes (Fig. 5A) to validate the transcriptome data. The results are consistent with those obtained with the microarrays (data not shown). All the transcriptome data have been submitted to GEO, accession number: GSE101411.

### *ΔspaF* mutant treatment alters *Desulfovibrio* and *Streptococcus* populations in the colon

**Figure 6:**
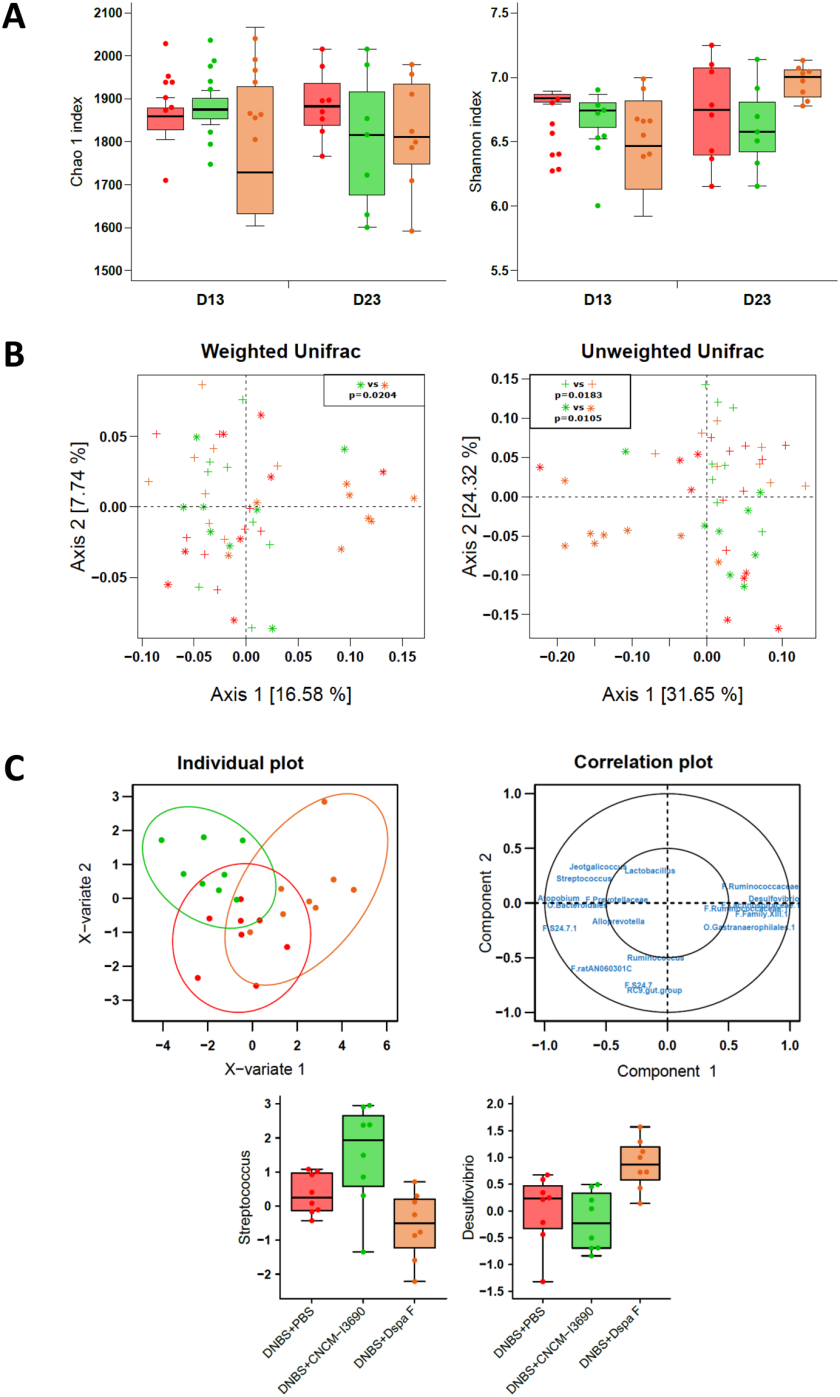
Microbiota analysis. Alpha diversity (Chao1 estimators and Shannon indices) was analyzed using a repeated measures two-way ANOVA on the D0 and D13 values, and a one-way ANOVA on the D13-adjusted D23 values (**A**). OTU data were examined using a principal coordinate analysis of weighted and unweighted UniFrac distances, and group effects were evaluated at each time point using a permutational ANOVA (adonis function in R). Post-hoc tests used Bonferroni corrections (**C**). Data were also analyzed at the genus level. They were filtered (retained if more than 60% non-zeros in at least one group and mean above 0.01% in at least one group). A PLS-DA was performed on the log ratios of abundance between D23 and D13 to discriminate among the three groups (two components). Individual plots with 95% confidence ellipses and a correlation plot are presented. Log ratios are plotted for the genera *Streptococcus* and *Desulfovibrio*. Mice groups are as in Fig. 3, with untreated mice in red, CNCM I-23690-treated mice in green, and *DspaF–treated*mice in orange; crosses and stars indicate D13 and D23 values, respectively (*n* = 8) (**C**).

Chronic DNBS-induced inflammation can induce changes in fecal microbiota, as measured using qPCR^26^. Here, though, low-grade inflammation did not result in microbiota shifts, as measured using 16 S sequencing (Fig. S2). Alpha diversity (Fig. S2A) and beta diversity (Fig. S2B) were not significantly different between the control and the untreated groups at any of the time points tested (Fig. S1). Among the three DNBS-challenged groups (Fig. 6), alpha diversity did not differ (Fig. 6A) from D13 to D23; however, beta diversity was different at D23. More specifically, the microbiota of the *ΔspaF–*treated group was distinct from that of the other two groups (Permanova test: p = 0.0116 and p = 0.0041 for weighted and unweighted UniFrac distances, respectively) (Fig. 6B). The taxonomic analyses of the three DNBS-challenged groups were performed by applying multivariate analysis (PLS-DA) to the log ratios of genus-level abundance between D13 and D23 (Fig. 6C). Eighteen variables were used to discriminate among the three groups; the clearest separation was between the CNCM I-3690-treated group and the *ΔspaF–*treated group. Interestingly, the *ΔspaF–*treated group had relatively less *Streptococcus* species and relatively more *Desulfovibrio* species compared to the two other groups (Fig. 6C). All the sequence data have been submitted to an ENA (European Nucleotide Archive) database, accession number: PRJEB22185.

## Discussion

The intestinal barrier plays a crucial role in homeostasis. The microbiota largely contributes to barrier integrity. Indeed, several preclinical studies have shown that some specific probiotic and commensal strains may improve mucosal barrier homeostasis. For instance, we have previously demonstrated that *L. rhamnosus* CNCM I-3690 can restore the integrity of the intestinal barrier. These results prompted us to explore the underlying bacterium-mediated mechanisms and host responses in this system^22^. To this end, we studied the two components of the intestinal barrier: (i) the physical layer composed of mucus and epithelial cells and (ii) the functional layer mainly composed of immune cells^16^.

A healthy intestinal barrier will allow selective paracellular transport. However, some diseases result in less controlled transport, thus uncoupling immune system activation and inflammation^27^. We previously found that CNCM I-3690 could counteract the *in vivo* increased permeability resulting from low-grade inflammation and modulate levels of the tight junction proteins occludin and E-cadherin^22^. Here, we determined that dysfunctional permeability was occurring at the colon and ileum levels and that CNCM I-3690 can also protect and/or restore the GC population, which is responsible for mucus production^28^. Besides, the transcriptome analysis revealed that CNCM I-3690 stimulates the expression of a gene typically expressed in GCs: the endogenous protease inhibitor Kazal-type 4 (Spink4)^29–31^. Endogenous protease inhibitors limit protease activity during homeostasis and inflammation^32^ and thus place breaks on the inflammatory cascade^32–34^. SPINKs could also protect against the proteolytic degradation of epithelial and mucosal tissues, and they are upregulated during bowel inflammation^29,35^. The transcriptome analysis also revealed that CNCM I-3690 upregulated the amino acid transporter SLC7A7. The resulting increased availability of amino acids can promote the growth of intestinal epithelial cells and improve absorption^36,37^.

As hyperpermeability increases local antigen exposure, it could also activate the intestinal immune response and provoke inflammation^27^. Here, we observed that low-grade inflammation altered the CD4^+^ T cell response, as previously reported^26,37,38^. We observed that the CNCM I-3690 treatment could counteract changes to the Th1/Th2 ratio as well as increase IL-10 production in both colon tissue and the supernatant of stimulated MLN and spleen cells. IL-10 is an important immunoregulatory cytokine that successfully suppresses the mucosal immune response associated with colonic inflammation^39^. Furthermore, IL-10 preserves the intestinal mucus barrier by suppressing protein misfolding and endoplasmic reticulum stress in GCs^40^.

Returning to the transcriptome analysis, we found that CNCM I-3690 upregulates three other hormones: ghrelin (GHR), peptide YY (PYY) and guanylate cyclase activator 2B (Guca2B). GHR is mostly produced in the stomach, but small amounts are generated in the small and large intestines^41^. At colonic level, it may accelerate motility and has been found to act as an anti-inflammatory factor, protecting the gut against a wide range of threats^42,43^. *In vitro*, GHR can restore the intestinal epithelium, inhibit pro-inflammatory cytokine expression and block the NF-κβ pathway^44–48^. Indeed, it improves TNBS-induced symptoms^49^. The peptide PYY regulates growth, digestion, and absorption^50^. Its role in colonic inflammation and dysfunction is controversial^51,52^.

GUCA2B is a ligand of Guanylate Cyclase 2 C (GUCY2C) receptor, which regulates ion secretion, intestinal barrier function and colonic mucosal inflammation^53,54^. Of note, GUCY2C^−/−^ mice suffer from restricted differentiation of GCs^55,56^ and disrupted GUCA2C signalling is associated with lower numbers of colonic GCs, resulting in decreased mucin production and reduced levels of tight-junction proteins, including occludin (a protein modulated by CNCM I-3690)^22^.

Here, we confirmed that CNCM I-3690 blocks canonical NF-κβ activation. However, the transcriptome analysis revealed that CNCM I-3690 upregulated the colonic expression of the TRAF-interaction protein with a forkhead-associated domain (TIFA). This adaptor can activate the NF-κβ canonical pathway via the TNF-α receptor-associated factor 6 (TRAF6)^57,58^. TIFA also activates TRAF2^59^. TRAF2 plays a cytoprotective role, enhance the survival of colonic epithelial cells and reduces inflammation in DSS-induced and spontaneous colitis, suggesting it is involved in the negative feedback process that restricts inflammation^60–63^. Nevertheless, this mechanism remains hypothetical and needs to be explored in future research. A putative mechanism of action of CNCM I-3690 is proposed in Fig. S3.

Since one of CNCM I-3690’s major probiotic effectors could be an extracellular structure helping to establish spatial proximity of bacteria with the epithelium we identified the strain’s range of adhesion proteins. It is known that pili structures can impact both adhesion and immune-related effects in some probiotics and commensals, we decided to inactivate CNCM I-3690 sole pili operon: *spaFED*^5,64,65^. Using an *in vitro* approach, we saw that the inactivation of the *spaF* gene resulted in the loss of the strain effects. Similarly, mice with induced low-grade inflammation treated with the *ΔspaF* mutant did not fully recover as when treated with the WT: their colonic permeability, colonic cytokine levels, GC populations, and lymphocyte populations remained altered.

The colonic transcriptome analysis revealed that the *ΔspaF* treatment changed the expression of 89 genes, 5 of which were also modulated by the CNCM I-3690 WT. Among the genes only modulated by the CNCM I-3690 WT was *tifa*. It is worth noting that the *ΔspaF* mutant was unable to block the NF-κβ pathway, supporting the hypothesized role of this signaling adaptor above. Among the genes modulated by the *ΔspaF* mutant, the most notable were the genes associated with G-protein-coupled receptor signaling (including cAMP-related signaling), ERK/MAPK signaling, and phospholipase C signaling. These pathways may be involved in colonic inflammation and cell proliferation^66–68^. This fact, combined with the absence of TIFA upregulation, could explain the failure of the *ΔspaF* mutant to block inflammation.

We observed that mice treated with the *ΔspaF* mutant had different microbiota than mice treated with the WT strain; namely, there was an increase in *Desulfobivrio* species and a decrease in *Streptococcus* species. *Desulfovibrio* is the most abundant genus of commensal sulfate-reducing bacteria (SBR) in the human colon^69^. *Desulfovibrio* species are capable of producing hydrogen sulphide (H_2_S), a gas with potentially genotoxic effects, by metabolizing dietary sulfites and sulfates as well as sulphomucins^70^. This genus displays increased prevalence in humans with ulcerative colitis (and other diseases involving colon inflammation) as well as in DSS-challenged mice, a pattern that is correlated with reduced mucosal thickness^70–73^. These results might indicate the importance of pili structure on the microbial environment of *L. rhamnosus* strain and its potential role in the interaction with other bacteria in the gut.

Taken together, our results confirm CNCM I-3690 probiotic potential for treating and/or preventing syndromes related to gut barrier dysfunction. In this study, we have specifically determined how CNCM I-3690 may provide benefits to its host and identified one of the major bacterial effectors involved.

## Methods

### Bacterial strains, cell lines, and culture conditions

*L. rhamnosus* CNCM I-3690 wild type (WT), the isogenic *DspaF* mutant and the complemented strain were cultured in MRS medium (Difco, USA) at 37 °C under aerobic conditions. Erythromycin (final concentration of 1 µg/ml) or chloramphenicol (final concentration of 10 µg/ml) (Sigma-Aldrich, Switzerland) was added as necessary.

The human cell lines Caco-2 (ATCC, UK), NF-κβ/SEAPorter HEK 293 (Imgenex, France), and HT-29 (ATCC) were grown in Dulbecco’s modified Eagle’s minimum essential medium (DMEM; Invitrogen, USA) supplemented with 25 mM glucose, 10% inactivated fetal bovine serum (FBS) (Lonza, France), 1% penicillin streptomycin (PS) and 1% glutamine (Invitrogen, France). For Caco-2 cells, media was supplemented also with 1% non-essential amino acid solution (Invitrogen).

### Identifying adhesins used by *L. rhamnosus* CNCM I-3690

A total of 105 bacterial genomes, including those of 23 *Lactobacillus* species, were examined (Table S3). Predicted nucleotide and protein sequences were obtained from the National Center for Biotechnology Information (NCBI; http://www.ncbi.nlm.nih.gov/) and a local MicroScope database hosted by Genoscope (https://www.genoscope.cns.fr/).

We compiled a non-exhaustive list of 63 proteins involved in adhesion: 12 pilins, 2 fimbrial adhesins (FAs), 11 fibronectin-binding proteins (FBPs), 10 choline-binding proteins (CBPs), and 28 mucus-binding proteins (MBPs) (Fig. 1). They were identified and analyzed using CLC DNA Workbench software (CLC bio, Denmark) for BLASTP analysis (default parameters)^74^. To eliminate proteins with partial domain matches, we used a 75% sequence identity threshold and required coverage of at least 75% of the query sequence length. Using BLASTP, we analyzed the distribution of different adhesin types in each genome.

### Construction of the *ΔspaF* mutant and the Δ*spaF* + *spaFDE* complementation strain

We amplified an internal fragment of the *spaF* gene (966 base pairs [bp]) via PCR using the proofreading ISIS-Taq polymerase (MP-Biomedicals) in accordance with the manufacturer’s instructions; primers OFF4045 (CTCAGCAAGCGATCTTGA) and OFF4046 (ATCTTGGCTAACCGCATC) used DNA from the CNCM I-3690 WT as the template. The fragment was cloned into the *Eco*RV-restriction site of the pOri280 plasmid (pDN0117 plasmid). Using electroporation, we introduced pDN0117 into a CNCM I-3690 strain carrying the temperature-sensitive plasmid pGhost3, which provides *repA* in trans for the conditional replication of pDN0117. Selection was performed under anaerobiosis at 30 °C on medium containing 2 µg/mL erythromycin. Integration of pDN0117 was obtained by increasing the temperature to 40 °C (2 µg/mL erythromycin; anaerobiosis). The result was the *DspaF* mutant.

To obtain the complementation strain, the whole *spa* locus was PCR amplified with the proofreading ISIS-Taq polymerase (MP-Biomedicals) in accordance with the manufacturer’s instructions and using the primers OFF4550 (AAGCTTAGGCACATAATGCTCATA) and OFF4541 (CTTATGACAAGCTCGAGGATTTA). The resulting 7357-bp fragment was cut with *Xho*I and *Sac*I restriction enzymes and cloned into pGhost3 digested with the same enzymes. The resulting plasmid, pDN142, was introduced into the *DspaF* mutant to obtain the *DspaF* + *spaFDE* strain (selection at 30 °C on 10 µg/mL chloramphenicol medium).

### *In vitro* immunomodulation, gut permeability, and adhesion assays

We performed *in vitro* assays of anti-inflammatory responses, GIT permeability, and bacterial adhesion as previously described^37,75,76^. Although classical probiotic strains are supposed to transit and no colonize, due to the potential benefices that the ability to adhere can confer to the strain we have also analyze their adhesion properties to mucus and epithelial cell lines as previously described^37,75,76^.

### Mouse model

Specific-pathogen-free (SPF) male C57BL/6 mice (Janvier, France) were housed in animal care facilities at the National Institute of Agricultural Research (INRA, IERP, Jouy-En-Josas, France) for at least one week before the induction of gut dysfunction^37^. Briefly, low-grade inflammation was generated by giving the mice two intrarectal injections of DNBS (100 mg/kg and 50 mg/kg, respectively; ICN, Biomedical Inc.) 21 days apart (Fig. S2A). In the control group, mice received vehicle injections.

Thirteen days after the first injection (i.e., D13), all mice received a 10-day gavage treatment. PBS (200 µl) was given to the control and one of the DNBS-challenged groups (hereafter, the untreated group). The two other groups were treated with 5 × 10^9^ CFU of viable bacteria in PBS (200 µl); one was given the CNCM I-3690 WT (hereafter, the CNCM I-3690-treated group) and the other was given the Δ*spaF* mutant (hereafter, the Δ*spaF*-treated group). All experiments were performed in accordance with EU animal care regulations and were approved by the relevant institutional committee (COMETHEA; protocol #02550.01).

We measured weight loss, colonic macroscopic scores, cytokine concentrations, serotonin concentrations, and myeloperoxidase (MPO) activity (a marker of polymorphonuclear neutrophil infiltration) as previously described^22,37,38^. Histological features were analyzed using hematoxylin-eosin-saffron (HES) staining, Alcian blue (AB) staining, and the periodic acid-Schiff (PAS) method in accordance with standard protocols^17,77^.

### Immunohistochemical analysis

To detect mucin 2 (MUC2), Carnoy-fixed samples were cut into 5-µm-thick sections, mounted on adhesive microscope slides (SuperFrost Ultra Plus, Thermo Scientific), and rehydrated and rinsed in accordance with standard protocols^17,77^. The samples were confined (Dako Pen, Agilent Technologies) and incubated sequentially with a protein block (Dako, Agilent Technologies), a primary antibody (2 μg/mL of MUC2 rabbit polyclonal IgG, Santa Cruz Biotechnologies), and a secondary antibody (2 ng/mL of Alexafluor 568 goat red anti-rabbit IgG, Invitrogen, Thermo Fischer Scientific); both of the antibodies were diluted (Dako Diluent, Agilent Technologies). Sections were then treated with trihydrochloride trihydrate (0.5 mg/mL Hoechst 33342, Invitrogen, Thermo Fischer Scientific) in PBS. The slides were mounted using fluorescent mounting medium (Dako, Agilent Technologies). Tissues were visualized using a high-capacity digital slide scanner (3DHISTECH Ltd.) and Pannoramic Viewer and CaseViewer software (3DHISTECH Ltd.)

### *In vivo* gut permeability assay

At the end of the probiotic experiment, permeability was determined *in vivo* using fluorescein-conjugated dextran (FD4 [3000–5000 Da], Sigma-Aldrich) as a tracer as previously described^78^.

Paracellular pathway permeability was measured using the flow of FD4 through colon and ileum samples, which were opened along the mesenteric border and mounted in Ussing chambers (P2300, Physiologic Instruments, USA). At 37 °C, 0.2 cm2 of tissue surface was exposed to 2.5 ml of 10 mM oxygenated Krebs-glucose and 10 mM Krebs-mannitol (serosal and luminal sides, respectively). FD4 (0.4 mg/ml) was added to the mucosal chamber, and samples were collected from the serosae chamber every 15 min for 2 h. FD4 concentrations were measured as described above.

### Colonic transcriptome analysis

Total RNA was isolated from colon samples (20–30 mg) using the RNeasy Mini Kit (Qiagen)^22^. RNA quantity was determined using a NanoDrop spectrophotometer, and RNA integrity was confirmed with an Agilent 2100 Bioanalyzer. The microarray analyses were carried out at the aBridge experimental facility (INRA, Jouy en Josas). We used a complete dye-swap reference design with five biological replicates, and we employed six SurePrint G3 8 × 60 K v2 microarrays (Agilent Technologies, France). Raw data were extracted from the microarray images using Agilent’s Feature Extraction software and preprocessed using the R package *agilp* downloaded from Bioconductor (http://www.bioconductor.org). More than 40% of the samples had undetected probes and were thus excluded from the analyses. Raw intensities were normalized using the *quantile normalization* method, and the resulting data were adjusted for batch effects using the ComBat method.

An empirical Bayesian test was used to analyze expression levels. Significant genes were identified by filtering based on adjusted *p-*values (using a threshold alpha of 0.05 and the Benjamini and Hochberg procedure for multiple comparisons). We then filtered based on expression levels, using |(FC)| > 1.25 as a cut-off^79^. For the remaining genes, ingenuity pathway analysis (IPA) was applied to log ratios and *p-*values to identify important pathways and generate data displays.

### Reverse transcription (RT) and quantitative real-time PCR (qPCR)

One μg of total RNA was reverse transcribed using an Applied Biosystems High-Capacity cDNA Reverse Transcription Kit (Thermo Fisher, France). The quantity of cDNA was determined using a NanoDrop spectrophotometer (Thermo Fisher). Quantitative real-time PCR (qPCR) was performed using duplicates of diluted cDNA (10-fold) and a StepOnePlus System (Applied). The reaction mix consisted of 12.5 μl of RoxSybr Master Mix blue dTTP (Takyon, Eurobio, France), 1 μl of each primer, and 1 μl of diluted cDNA, all in a final volume of 25 μl. For the validation of the transcriptome results, primers were purchased from Qiagen (RT2 qPCR Assay). Values were expressed as relative-fold differences using a housekeeping gene, *Gapdh*, as a standard; we employed the 2^−ΔΔCT^ method. All procedures were performed in accordance with the manufacturers’ instructions.

### Analyses of lymphocyte populations

We obtained mononuclear cells via the gentle extrusion of tissue from the spleen and the mesenteric lymph nodes (MLNs); the cells were analyzed using flow cytometry (Accuri, BD) and CFlow Sampler software (BD Biosciences) as described previously^37^. Briefly, 1 × 10^6^–10^7^ cells were labeled with anti-CD3 FITC, anti-CD4 PerCP, anti-T-bet APC, and anti-GATA3-PE (all from eBioscience).

We performed stimulation experiments in which 2 × 10^5^ cells per well were stimulated with anti-CD3/CD28 antibodies (eBioscience, San Diego, USA) as described previously^37^. We determined supernatant cytokine concentrations using a cytometric bead array system (Mouse Th1/Th2/Th17/Th22 13-Plex FlowCytomix Multiplex; eBioscience) in accordance with the manufacturer’s instructions.

### Intestinal microbiota sequencing and analysis

A total of 96 fresh fecal samples were collected from all four groups in the probiotic experiment at three time points (D0, D13, and D23; Fig. S1A) and stored at −80 °C. DNA was then obtained via mechanical lysis (Fastprep® FP120 [ThermoSavant]) and phenol/chloroform-based extraction as described previously^80^. Amplification was performed using the V3-V4 primers for 16 s rRNA (forward: CCTACGGGNGGCWGCAG, reverse: GACTACHVGGGTATCTAATCC)^81^. The samples were loaded into flow cells in an Illumina MiSeq. 300PE Sequencing Platform in accordance with the manufacturer’s instructions. Analyses were performed using QIIME (v. 19). After filtering for quality, a mean of 85,073 ± 24,497 sequences per sample were retained. Reads were clustered into operational taxonomic units (OTUs; 97% identity threshold) using VSEARCH, and representative sequences for each OTU were aligned and taxonomically assigned using the SILVA database (v. 119). To characterize diversity, rarefaction was used to obtain 30,000 sequences per sample. Alpha diversity (within samples) was represented using total species richness (Chao1 estimator) and evenness (Shannon index). Beta diversity (between samples) was represented using UniFrac distances calculated from OTU counts. Statistical analyses were performed using SAS 9.3 and R 3.3.0 (vegan and mixOmics packages^82,83^).

### Statistical Analysis

Except for microbiota and trascriptome analysis, statistical analyses were performed using GraphPad software (GraphPadSofware, La Jolla, CA, USA). We carried out non-parametric Kruskal-Wallis tests followed by Dunn’s multiple comparison tests. *P*-values below 0.05 were considered significant.

## Supplementary information

Supplementary data
