# Supplementary material for: The potential probiotic Lactobacillus rhamnosus CNCM I-3690 strain protects the intestinal barrier by stimulating both mucus production and cytoprotective response
Source: Sci Rep. 2019 Apr 1;9:5398. doi: 10.1038/s41598-019-41738-5 (PMC6443702; doi:10.1038/s41598-019-41738-5)
Supplement: Supplementary file 1 — Supplementary data [file 41598_2019_41738_MOESM1_ESM.pdf]

The potential probiotic *Lactobacillus rhamnosus* CNCM I-3690 strain protects the intestinal barrier by stimulating both mucus production and cytoprotective response

Rebeca Martín[1]; Celia Chamignon[1]; Nadia Mhedbi-Hajri[2]; Florian Chain[1]; Muriel Derrien[2]; Unai Escribano-Vázquez[1]; Peggy Garault[2]; Aurélie Cotillard[3]; Hang Phuong Pham[4]; Christian Chervaux[2]; Luis G. Bermúdez-Humarán[1]; Tamara Smokvina*[2]; Philippe Langella*[1].

[1]INRA, Commensal and Probiotics-Host Interactions Laboratory, Micalis Institute, INRA, AgroParisTech, Université Paris-Saclay, 78350 Jouy-en-Josas, France; [2]Danone Nutricia Research, Av de la Vauve, 91767, Palaiseau, France. [3]Soladis, Paris, France. [4]ILTOO Pharma, 14 Rue des Reculettes, 75013, Paris, France.

Rebeca.martin-rosique@inra.fr , Celia.chagminon@inra.fr , nadiahajrimhedbi@hotmail.com ,

Florian.chain@inra.fr , Muriel.DERRIEN@danone.com , unai.escribano-vazquez@inra.fr ,

Peggy.GARAULT@danone.com , Aurelie.COTILLARD@external.danone.com ,

hp.pham@iltoopharma.fr , Christian.CHERVAUX@danone.com , luis.bermudez@inra.fr ,

Tamara.smokvina@danone.com , Philippe.langella@inra.fr

Running title: *Lactobacillus rhamnosus* protects intestinal barrier

*Corresponding authors

ADDITIONAL FILES

Figure S1. Experimental protocols for the mouse model of chronic micro-inflammation and evaluation of inflammatory status of DNBS-challenged mice. Colitis was induced by intrarectal administration of 100 mg/Kg of DNBS in solution in 25% ethanol (EtOH). Control mice (without colitis) received only the vehicle. The effects of DNBS are highest during the first 3 days after its administration (DNBS period). Ten days after the end of the DNBS period bacterial culture or PBS were intragastrically administered daily for 10 days (gavage period). Colitis was reactivated 21 days after the first DNBS injection with a second injection of 50 mg/Kg of DNBS solution. Three days after reactivation mice were sacrificed. Inflammatory status assessed from (A) the recovery after reactivation (gr); (B) macroscopic score; (C,D) colon or ileum MPO activity and E) colonic cytokine concentrations (n=30 mice per group) in the control group (vehicle+PBS), untreated group (DNBS+PBS), *L. rhamnosus* CNCM I-23690 strain (DNBS+CNCM I-3690) and *L. rhamnosus DspaF*strain group (DNBS+□spaF). *$p < 0.05$ **$p < 0.01$. (n=30)

Figure S2. Effect of low-grade inflammation DNBS model on host microbiota. Chao1 and Shannon indices were analysed using a non-parametric Mann-Whitney test at D0, and then a repeated-measures two-way anova adjusted by D0 values for D13 and D23 (A). OTUs data were visualized using principal coordinate analysis on weighted and unweighted Unifrac distances, and group effects were evaluated at each time point using permutational anova (adonis function in R) (B). Blue: control group (vehicle-PBS), red: untreated group (DNBS-PBS), dots: D0, crosses D13, Starts:D23. (n=8)

Figure S3. Putative mechanism of action of CNCM I-3690. When CNCM I-3690 arrives into the colon, it is able to attach to the mucus and the epithelial cell surface allowing a tight crosstalk with the host. CNCM I-3690 upregulates the expression of GUCA-2B which (through its interaction with GUCY-2C) will enhance the differentiation of GC and the restoration of tight

junction proteins (mainly occludin). GC will increase the production of MUC2 and of the protective inhibitor of peptidases SPINK4 allowing the development of a thickness mucus layer. Furthermore, an increase of the amino acid transporter SLC7A7 will allow the proliferation of the epithelium. CNCM I-3690 increases the expression of Ghrelin (GHR) and polypeptide Y (PYY), with a positive direct effect on serotonin production and motility. Furthermore, CNCM I-3690 also counterbalances the Th1/Th2 ratios and increase IL-10 production maybe mediated by GHR. Finally, CNCM I-3690 increases TIFA expression, an adaptor protein able to bind TRAF-2, which could induce the activation of the non-canonical NF-KB and the block of the inflammatory process. Thanks to all these interactions, the barrier returns to normal and the micro-inflammation and gut dysfunction status disappear.

Table S1. Colonic transcriptome analysis of mice treated with *D*SpaF I. Genes up regulated by the *D*SpaF strain versus DNBS-PBS group.

Table S2. Colonic transcriptome analysis of mice treated with *D*SpaF II. Genes down regulated regulated by the *D*SpaF strain versus DNBS-PBS group.

Table S3. List of *Lactobacillus* strains used in the study for the genomic analyses.

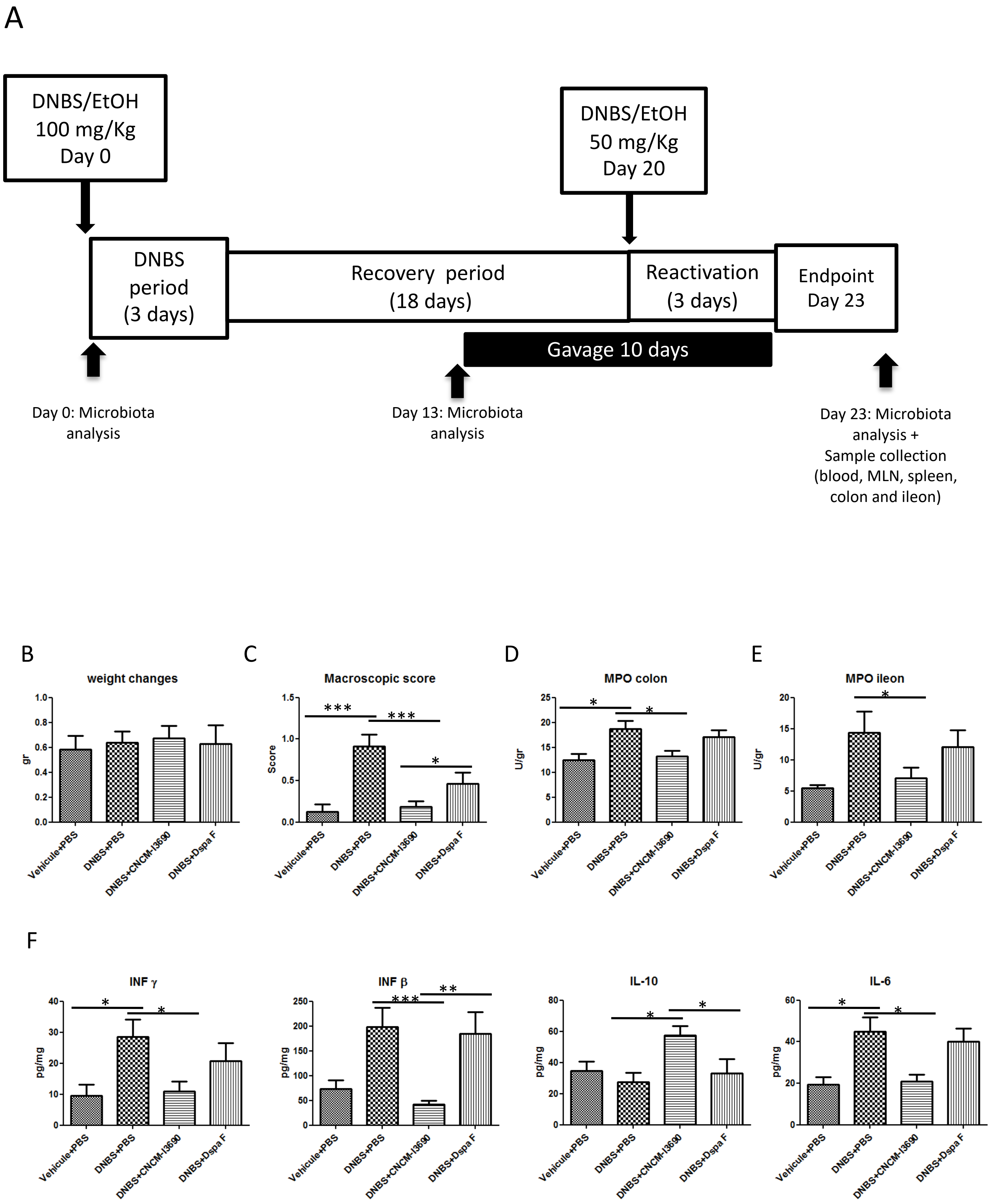
A
DNBS/EtOH
100 mg/Kg
Day 0
DNBS/EtOH
50 mg/Kg
Day 20
DNBS
period
(3 days)
Recovery period
(18 days)
Reactivation
(3 days)
Endpoint
Day 23
Gavage 10 days
Day 0: Microbiota analysis
Day 13: Microbiota analysis
Day 23: Microbiota analysis + Sample collection (blood, MLN, spleen, colon and ileon)
B
weight changes
gr
C
Macroscopic score
Score
D
MPO colon
U/gr
E
MPO ileon
U/gr
Vehicule+PBS
DNBS+PBS
DNBS+CNCM-I3690
DNBS+Dspa F
F
INF γ
INF β
IL-10
IL-6
pg/mg

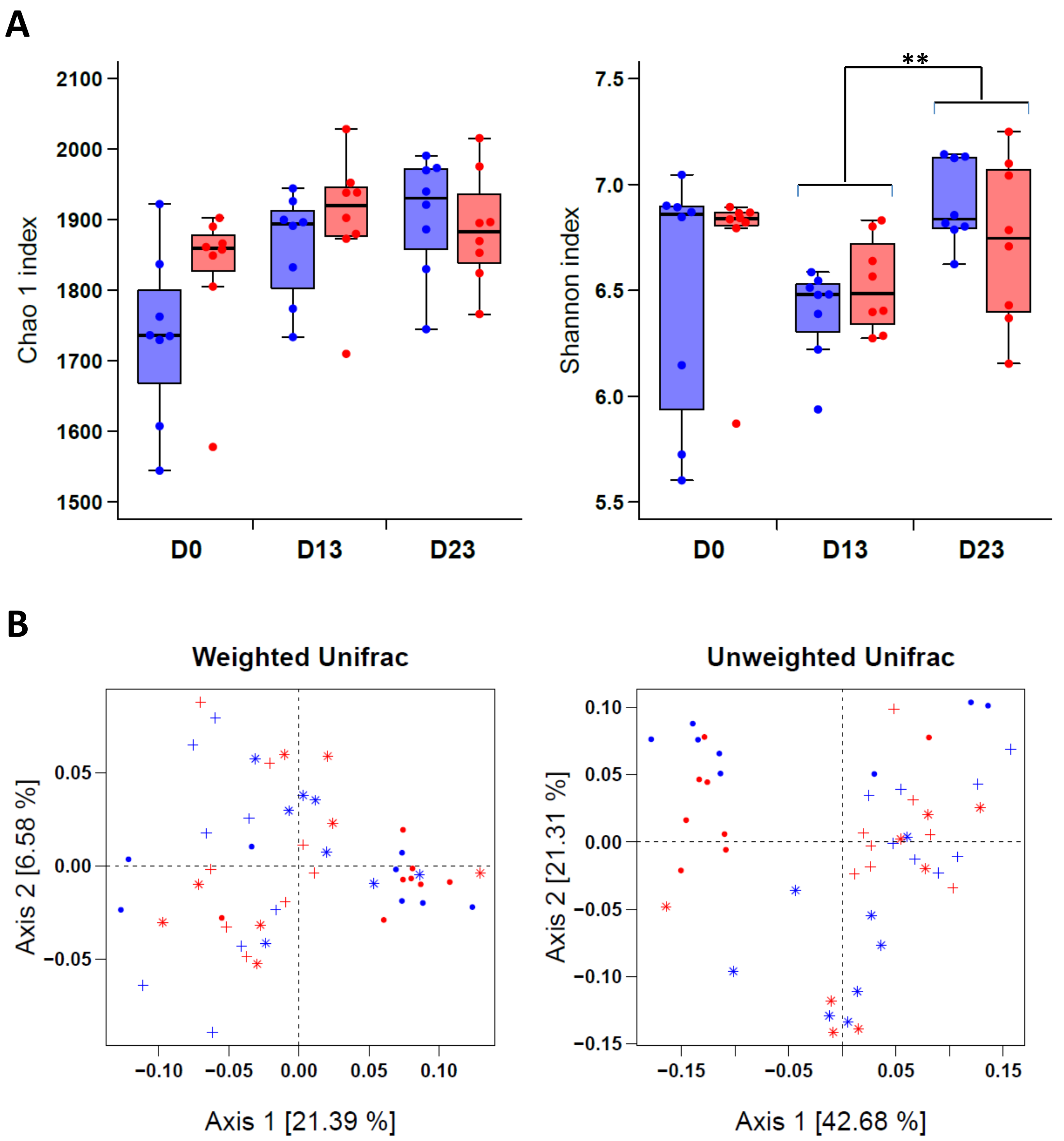
A
Chao 1 index
2100
2000
1900
1800
1700
1600
1500
D0
D13
D23
Shannon index
7.5
7.0
6.5
6.0
5.5
**
B
Weighted Unifrac
Axis 2 [6.58 %]
Axis 1 [21.39 %]
0.05
0.00
−0.05
−0.10
0.10
Unweighted Unifrac
Axis 2 [21.31 %]
Axis 1 [42.68 %]
−0.15
0.15

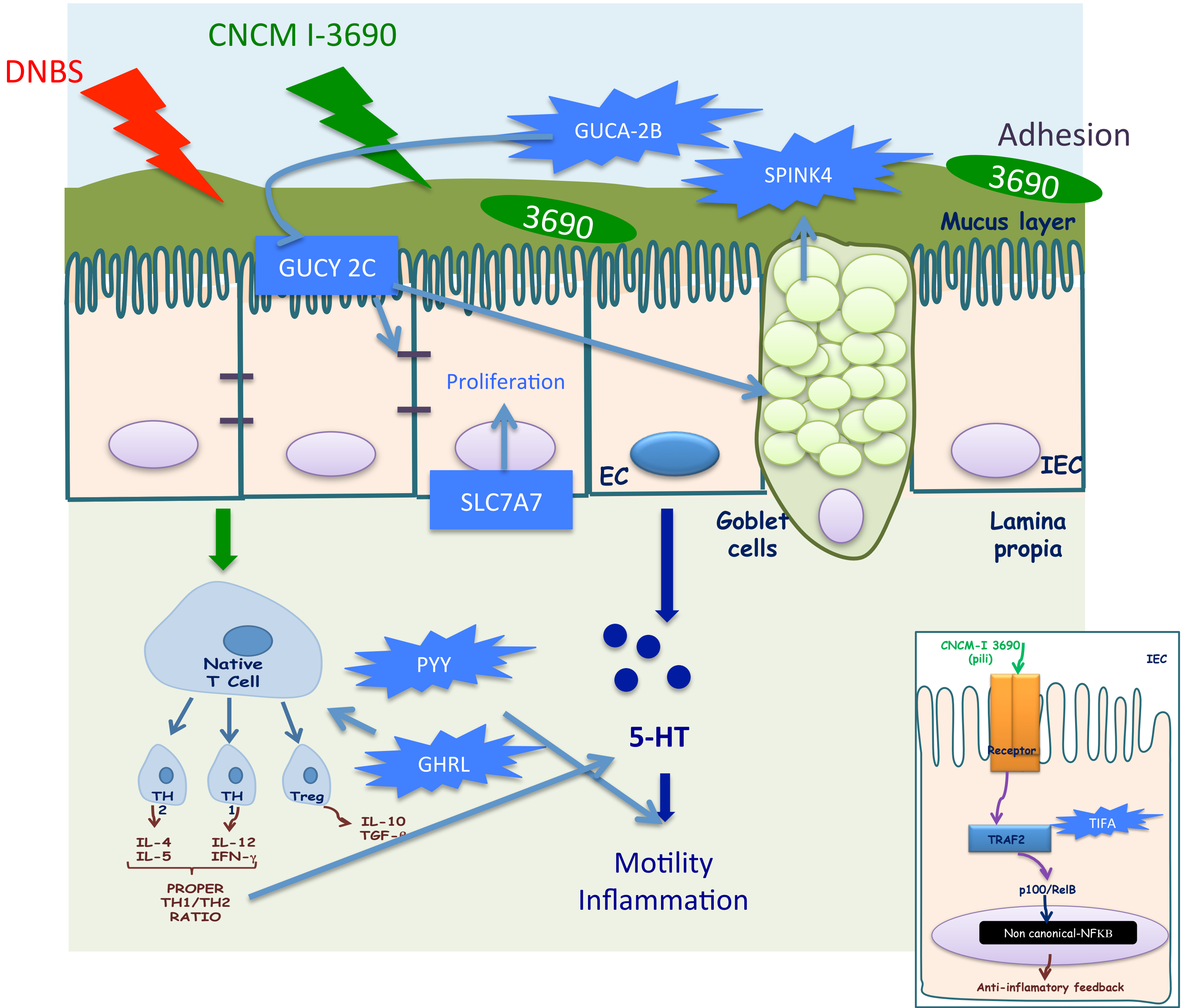

CNCM I-3690
DNBS
GUCA-2B
SPINK4
Adhesion
3690
3690
Mucus layer
GUCY 2C
Proliferation
SLC7A7
EC
IEC
Goblet cells
Lamina propia
Native T Cell
PYY
GHRL
TH 2
TH 1
Treg
IL-10
IL-4
IL-5
IL-12
IFN-γ
PROPER TH1/TH2 RATIO
5-HT
Motility
Inflammation
CNCM-I 3690 (pili)
IEC
Receptor
TRAF2
TIFA
p100/RelB
Non canonical-NFKB
Anti-inflamatory feedback

Table S1. Colonic transcriptome analysis of mice treated with *D*SpaF. Genes up regulated by the *D*SpaF strain versus DNBS-PBS group.

FC	Gene	Description
3,038	D630010B17Rik	Mus musculus RIKEN cDNA
3,031	Wfdc18	Mus musculus WAP four-disulfide core domain 18 (Wfdc18)
3,009	Myl7	Mus musculus myosin, light polypeptide 7
2,979	Kcnh3	Mus musculus potassium voltage-gated channel, subfamily H (eag-related), member 3
2,820	Ghrl	Mus musculus ghrelin
2,604	Spink4	Mus musculus serine peptidase inhibitor, Kazal type 4 (Spink4)
2,534	Scgb2b2	Mus musculus secretoglobin, family 2B, member 2 (Scgb2b2)
2,377	Fam64a	Mus musculus family with sequence similarity 64, member A
2,282	Stc2	Mus musculus stanniocalcin 2 (Stc2)
2,087	Fam167b	Mus musculus family with sequence similarity 167, member B
2,080	Col6a5	Mus musculus collagen, type VI, alpha 5
2,019	Scgb2b20	Mus musculus secretoglobin, family 2B, member 20
1,980	Slc12a8	Mus musculus solute carrier family 12 (potassium/chloride transporters), member 8
1,927	Scgb2b15	Mus musculus secretoglobin, family 2B, member 15 (Scgb2b15),
1,924	Ccdc109b	Mus musculus coiled-coil domain containing 109B
1,890	Ust	Mus musculus uronyl-2-sulfotransferase
1,865	Rapgef3	Mus musculus Rap guanine nucleotide exchange factor (GEF) 3
1,858	Exoc3l4	Mus musculus NOD-derived CD11c +ve dendritic cells cDNA
1,858	Ptpro	Mus musculus adult male corpora quadrigemina cDNA
1,819	Mrgpra2a	Mus musculus MAS-related GPR, member A2A
1,794	Rap1gapos	PREDICTED: Mus musculus RAP1 GTPase activating protein, opposite strand
1,774	Fscn1	Mus musculus fascin homolog 1, actin bundling protein (Strongylocentrotus purpuratus)
1,730	Myef2	Mus musculus myelin basic protein expression factor 2, repressor
1,715	Kif4	kinesin family member 4
1,709	Pyy	Mus musculus peptide YY
1,677	Guca2b	Mus musculus guanylate cyclase activator 2b
1,664	Il3ra	Mus musculus interleukin 3 receptor, alpha chain
1,656	Smoc2	Mus musculus SPARC related modular calcium binding 2
1,628	Creb3l4	Mus musculus cAMP responsive element binding protein 3-like 4
1,625	Ntn5	Mus musculus netrin 5
1,602	Gfra3	Mus musculus glial cell line derived neurotrophic factor family receptor alpha 3
1,581	Cpa2	carboxypeptidase A2, pancreatic [
1,547	Aqp5	Mus musculus aquaporin 5
1,536	Gpr20	Mus musculus G protein-coupled receptor 20 (Gpr20)
1,533	Dll3	Mus musculus delta-like 3 (Drosophila) (Dll3)

1,533	Txndc5	Mus musculus thioredoxin domain containing 5
1,522	Rap1gap	Mus musculus adult male eyeball cDNA
1,467	Fes	Mus musculus feline sarcoma oncogene
1,466	Unc5a	Mus musculus unc-5 homolog A (C. elegans)
1,462	Galnt12	Mus musculus UDP-N-acetyl-alpha-D-galactosamine:polypeptide N-acetylgalactosaminyltransferase
1,462	Pla2g2f	Mus musculus phospholipase A2, group IIF
1,444	2700094K13Rik	Mus musculus RIKEN cDNA 2700094K13 gene
1,430	Rgs14	Mus musculus regulator of G-protein signaling 14
1,387	Trim46	Mus musculus tripartite motif-containing 46 (Trim46)
1,371	Kif12	Mus musculus kinesin family member 12
1,370	Hyal5	Mus musculus hyaluronoglucosaminidase 5
1,366	Slc7a7	Mus musculus solute carrier family 7 (cationic amino acid transporter, y+ system), member 7
1,366	Prc1	Mus musculus protein regulator of cytokinesis 1
1,366	Twf2	Mus musculus twinfilin, actin-binding protein, homolog 2 (Drosophila)
1,363	1700010I14Rik	Mus musculus RIKEN cDNA 1700010I14 gene
1,356	Rnf26	Mus musculus ring finger protein 26
1,284	Gm9099	Mus musculus 3 days neonate thymus cDNA
1,271	Pepd	Mus musculus peptidase D

Table S2. Colonic transcriptome analysis of mice treated with *D*SpaF. Genes down regulated regulated by the *D*SpaF strain versus DNBS-PBS group.

FC	Gene	Description
0,750	Ctss	Mus musculus cathepsin S
0,739	Arhgap12	Mus musculus Rho GTPase activating protein 12
0,737	B230214O09Rik	Mus musculus adult male corpora quadrigemina cDNA,
0,733	Atp2b1	Mus musculus ATPase, Ca++ transporting, plasma membrane 1
0,732	Gm31222	PREDICTED: Mus musculus predicted gene, 31222
0,722	Slk	Mus musculus STE20-like kinase (Slk), transcript variant 1
0,700	Gm40798	Mus musculus 3 days neonate thymus cDNA
0,700	Cpvl	Mus musculus carboxypeptidase, vitellogenic-like
0,692	Lhfpl2	Mus musculus lipoma HMGIC fusion partner-like 2
0,671	Rmi1	Mus musculus RMI1, RecQ mediated genome instability 1, homolog (S. cerevisiae)
0,661	Wwp2	Mus musculus 0 day neonate eyeball cDNA
0,645	Gm36204	Mus musculus predicted gene, 36204
0,640	Mtm1	X-linked myotubular myopathy gene 1
0,639	Arrdc3	arrestin domain containing 3
0,635	Lce1c	Mus musculus late cornified envelope 1C
0,634	Btnl10	Mus musculus butyrophilin-like 10
0,626	C80012	H3045G09-5 NIA Mouse 15K cDNA Clone Set Mus musculus cDNA clone H3045G09 5'
0,622	Phlpp2	Mus musculus PH domain and leucine rich repeat protein phosphatase 2
0,622	Plekha6,	Mus musculus pleckstrin homology domain containing, family A member 6
0,621	Pde7a	Mus musculus phosphodiesterase 7A
0,619	Gm12576	Mus musculus 0 day neonate cerebellum cDNA
0,617	Gm5433	Mus musculus 13 days embryo male testis cDNA
0,615	Irs2	Mus musculus insulin receptor substrate 2
0,614	Stk17b	Mus musculus serine/threonine kinase 17b (apoptosis-inducing)
0,610	Cebpd	Mus musculus CCAAT/enhancer binding protein (C/EBP), delta
0,610	Tnfaip3	Mus musculus tumor necrosis factor, alpha-induced protein 3
0,603	Mical1	Mus musculus microtubule associated monooxygenase, calponin and LIM domain containing 1
0,572	Rasgef1b	Mus musculus RasGEF domain family, member 1B
0,555	Ccrn4l	CCR4 carbon catabolite repression 4-like (S. cerevisiae)
0,538	Hs3st4	Mus musculus heparan sulfate (glucosamine) 3-O-sulfotransferase 4
0,536	6330412A17Rik	Mus musculus adult male medulla oblongata cDNA

Table S3. List of *Lactobacillus* strains used in the study for the genomic analyses.

Bacteria species	Strain	Sequence Status	Origin of the sequence
L. acidophilus	NCFM	Refseq	NCBI
	30SC	Refseq	NCBI
	ATCC 4796	Scaffolds or Contigs	NCBI
L. amylovorus	GRL1112	Refseq	NCBI
	GRL1118	Refseq	NCBI
L. brevis	ATCC367	Refseq	NCBI
	ATCC 27305	Scaffolds or Contigs	NCBI
L. buchneri	ATCC 11577	Scaffolds or Contigs	NCBI
	CD034	Refseq	NCBI
	NRRL B-30929	Refseq	NCBI
L. crispatus	214-1	Scaffolds or Contigs	NCBI
	CTV-05	Scaffolds or Contigs	NCBI
	FB049-03	Scaffolds or Contigs	NCBI
	FB077-07	Scaffolds or Contigs	NCBI
	ST1	Refseq	NCBI
L. delbruckiisubspsp. bulgaricus	2038	Refseq	NCBI
	ATCC BAA-365	Refseq	NCBI
	ATCC 11842	Refseq	NCBI
	CNCM I-2836	Contigs	Danone Research
	CNCM I-1632	Contigs	Danone Research
	CNCM I-1519	Contigs	Danone Research
	CNCM I-2787	Contigs	Danone Research
	ND02	Refseq	NCBI
	CNCM I-3741	Contigs	Danone Research
L. fermentum	28-3-CHN	Scaffolds or Contigs	NCBI
	ATCC 14931	Scaffolds or Contigs	NCBI
	CECT 5716	Refseq	NCBI
	IFO 3956	Refseq	NCBI
L. gasseri	202-4	Scaffolds or Contigs	NCBI
	224-1	Scaffolds or Contigs	NCBI
	ATCC 33323	Refseq	NCBI
	CECT5714	Scaffolds or Contigs	NCBI
	JV-V03	Scaffolds or Contigs	NCBI
	MV-22	Scaffolds or Contigs	NCBI
L. helveticus	CNCM I-3504	Scaffolds or Contigs	Danone Research
	DPC4571	Refseq	NCBI
	DSM 20075	Scaffolds or Contigs	NCBI
	H10	Refseq	NCBI
	MTCC 5463	Scaffolds or Contigs	NCBI
	R0052	Refseq	NCBI
L. jensenii	1153	Scaffolds or Contigs	NCBI

	115-3-CHN	Scaffolds or Contigs	NCBI
	269-3	Scaffolds or Contigs	NCBI
	27-2-CHN	Scaffolds or Contigs	NCBI
	JV-V16	Refseq	NCBI
L. johnsonii	ATCC 33200	Scaffolds or Contigs	NCBI
	DPC6026	Refseq	NCBI
	FI9785	Refseq	NCBI
	NCC 533	Refseq	NCBI
	pf01	Scaffolds or Contigs	NCBI
L. kefirnofaciens	ZW3	Refseq	NCBI
L. mucosae	CNCM I-4429	Contigs	Danone Research
L. paracasei	ATCC 334	Refseq	NCBI
	BD-II	Refseq	NCBI
	BL23	Refseq	NCBI
	LC2W	Refseq	NCBI
	W56	Refseq	NCBI
	Zhang	Refseq	NCBI
	CNCM I-1518	Contigs	Danone Research
	Lpp22	Contigs	Danone Research
	CNCM I-3689	Contigs	Danone Research
	Lpp193	Contigs	Danone Research
	Lpp197	Contigs	Danone Research
	Lpp217	Contigs	Danone Research
	Lpp17	Contigs	Danone Research
	Lpp46	Contigs	Danone Research
	Lpp120	Contigs	Danone Research
	Lpp225	Contigs	Danone Research
	Lpp226	Contigs	Danone Research
	8700:2	Scaffolds or Contigs	NCBI
L. pentosus	KCA1	Refseq	NCBI
L. plantarum	CNCM I-3436	Contigs	Danone Research
	CNCM I-4318	Contigs	Danone Research
	JDM1	Refseq	NCBI
	WCFS1	Refseq	NCBI
	ST-III	Refseq	NCBI
L. reuteri	100-23	Scaffolds or Contigs	NCBI
	DSM20016	Refseq	NCBI
	JCM1112	Refseq	NCBI
	MM4-1A	Scaffolds or Contigs	NCBI
	SD2112	Refseq	NCBI
L. rhamnosus	ATCC 8530	Refseq	NCBI
	CNCM I-3690	Contigs	Danone Research
	Lr9	Contigs	Danone Research
	CNCM I-2493	Contigs	Danone Research
	Lr52	Contigs	Danone Research

	Lr64	Contigs	Danone Research
	Lr74	Contigs	Danone Research
	Lr75	Contigs	Danone Research
	Lr108	Contigs	Danone Research
	Lr110	Contigs	Danone Research
	GG	Refseq	NCBI
	GG ATCC 53103	Refseq	NCBI
	HN001	Refseq	NCBI
	Lc 705	Refseq	NCBI
L. ruminis	ATCC 27782	Refseq	NCBI
	ATCC25644	Scaffolds or Contigs	NCBI
	SPM0211	Scaffolds or Contigs	NCBI
L. sakei	23K	Refseq	NCBI
L. salivarius	ATTC 11741	Scaffolds or Contigs	NCBI
	CECT 5713	Refseq	NCBI
	GJ-24	Scaffolds or Contigs	NCBI
	NIAS840	Scaffolds or Contigs	NCBI
	UCC118	Refseq	NCBI
L. sanfrancisensis	TMW1.1304	Refseq	NCBI
